# Laser Heterodyne Detection Based on Photon Time–Domain Differential Detection Avoiding the Effect of Decoherence Phase Noise

**DOI:** 10.3390/s23239435

**Published:** 2023-11-27

**Authors:** Ce Guan, Zijing Zhang, Fan Jia, Yuan Zhao

**Affiliations:** School of Physics, Harbin Institute of Technology, Harbin 150001, China; 19b911036@stu.hit.edu.cn (C.G.); zhangzijing@hit.edu.cn (Z.Z.); 19b911037@stu.hit.edu.cn (F.J.)

**Keywords:** photon time–domain differencing, phase noise, decoherence limit, photon-level echo

## Abstract

Laser heterodyne detection (LHD) is a key velocimetry technique that provides better accuracy and sensitivity than direct laser detection. However, random phase noise can be introduced by the surface topography of the moving target undulation or atmospheric turbulence during transmission. The random phase noise causes the target echo to undergo decoherence, resulting in degradation of the signal-to-noise ratio (SNR). Here, we propose a novel LHD method based on photon time–domain differential detection. It can infer the heterodyne spectrum of the target echo and the local oscillator light from the time intervals of the photon arrival. The time interval statistic is a relative quantity, which can effectively avoid the effect of random phase noise in LHD. With our method, the SNR of LHD can be improved in application scenarios where the target echo is decoherent. We developed a complete solution model for acquiring the heterodyne spectrum based on photon time–domain differential detection and performed proof-of-principle experiments. The experimental results show that in the presence of random phase noise, the SNR and velocity measurement error of our method are significantly better than that of the conventional method, and the larger the phase noise is, the more the SNR and velocity measurement error of our method are improved. Moreover, along with the increase in phase noise, the SNR of our method is basically unchanged, which also indicates that our method is not affected by random phase noise. This advantage is significant for photon-level weak echoes that require long detection times to be detected.

## 1. Introduction

Laser heterodyne detection (LHD) measures the interference signal between the target echo and the locally oscillating (LO) light, to obtain the frequency, phase, and amplitude information of the target echo and ultimately realize the measurement of the target distance, velocity, vibration, and so on [1,2]. LHD has an advantage over laser direct detection in terms of accuracy and sensitivity in acquiring the multidimensional motion information of a target [3,4,5,6,7]. In LIDAR application scenarios, such as synthetic aperture lidar and advanced remote sensing [8,9,10,11,12,13,14], etc., surface topography undulations of a moving target or atmospheric turbulence can lead to random phase fluctuations at different points in the optical field, thus introducing random phase noise [15,16]. The random phase noise eventually leads to decoherence of the target echo [8,17]. In this case, the random phase noise of the target echo will cause the heterodyne photocurrents in the conventional LHD method to cancel each other out [18,19]. The SNR of the conventional LHD method will be reduced. The random phase noise of the target echo will limit the detection sensitivity and velocimetry error of the conventional LHD method.

The current solution for this situation is to beam expand the target echo, use multiple detectors to simultaneously heterodyne detect the target echo in each part of the expanded beam, and compensate the intermediate frequency photocurrent generated by each detector through a post-processing algorithm [10,20,21,22,23,24,25]. Its core is to design the corresponding algorithm to compensate for the phase noise according to the wavefront distribution characteristics of the target echo. However, this method is effective in compensating and improving slow-varying phase noise such as atmospheric turbulence but is not ideal for fast-varying phase noise caused by undulation of the target surface topography due to the lateral motion of the target.

In this paper, a photon time–domain differential detection (PTDD) method is proposed for LHD, which is not affected by the fast-varying phase noise of the target echo. In our method, the target echo and the local oscillator (LO) light are interfered with and detected by two single-photon avalanche detectors (SPADs). The time interval data of the neighboring photon counting pulses (PCPs) generated by the SPADs are collected. The heterodyne spectrum can be obtained by Fourier transforming the time interval data. Finally, the velocity information of the target is obtained by analyzing the heterodyne spectrum. We developed a complete solution model for acquiring the heterodyne spectrum based on photon time–domain differential detection and performed proof-of-principle experiments. The experiments showed that the SNR of the conventional method by detecting the photocurrent drops to 0.4 times the original one with the increase in random phase noise, while the SNR of the photon time–domain differential detection method is only 0.9 times the original one, which is almost unchanged. Due to the reduced SNR, the velocity measurement error of the conventional method increases almost twice as fast as the method in this paper. Our method can improve the SNR and reduce the velocity measurement error of the LHD in atmospheric turbulence and the surface topography of the moving target undulation. This advantage is significant for photon-level weak echoes that require long detection times to be detected.

## 2. System Description and Method

**Figure 1:**
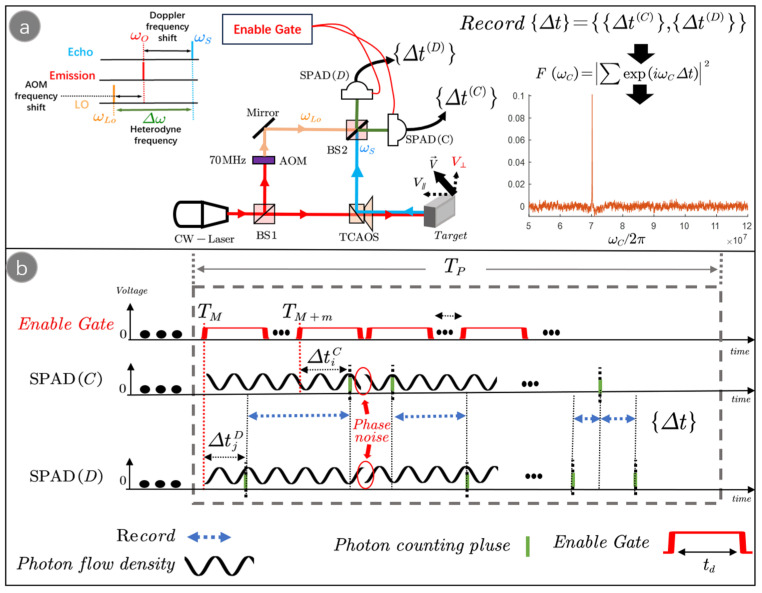
(**a**) Laser heterodyne detection system based on the photon time–domain differential detection method. SPAD is the single-photon avalanche detector, TCAOS is the transceiver common aperture optical system, BS is the beam splitter, and AOM is the acoustic-optic modulator. (**b**) Schematic diagram of the process of collecting time interval data through photonic time–domain differential detection.

As shown in Figure 1a, the optical frequency of the laser beam emitted from a continuous wave laser (CW-laser) is $\omega_{O}$. The laser beam is split into two beams by a beam splitter (BS1), one of which is modulated by an acoustic-optic modulator (AOM) to be used as LO light. The optical frequency of the LO light is changed by the AOM to $\omega_{Lo}$. The other laser beam is directed to the target surface topography undulation through the transceiver common aperture optical system (TCAOS), reflected by the target, and collected by the TCAOS as the target echo, which has an optical frequency $\omega_{S}$. The target echo and the LO light are detected by the SPADs after interfering with the BS2 (50/50), and the time interval data {Δt} are collected. The heterodyne spectrum $F(\omega_{C})$ can be obtained by Fourier transforming the time interval data {Δt}. Finally, the velocity information of the target is obtained by $\Delta \omega =\underset{\omega_{C}}{\arg\max}\left\{F(\omega_{C})\right\}$. Figure 1b shows the acquisition process of the time interval data. Under the enable gate, the SPADs generate PCPs at such moments as $T_{M}+\Delta t_{j}^{D}$, $T_{M+m}+\Delta t_{i}^{C}$. The system collects the time intervals of neighboring PCPs $\Delta t=T_{M+m}+\Delta t_{i}^{C}-(T_{M}+\Delta t_{j}^{D})$.

## 3. Solution Model for the Heterodyne Spectrum Based on the PTDD Method

Considering the fast-varying phase noise caused by undulation of the target surface topography due to lateral motion, let the target surface topography undulation change every $\tau_{s}$ during the detection process. For the whole detection process $T_{P}\leq n\tau_{s}$, it is considered that the relative phases between the target echo and the LO light, $\theta_{i},i=1,2,\cdots ,n$, are kept constant within $\tau_{s}$. The target echo light field $E_{S}(t)$ can be expressed as:

(1) $$E_{S}(t)=\left\{\begin{array}{cc} A_{S}\exp[i\omega_{S}t+\theta_{1}]{\vec{e}}_{S}, & \;\;\;\;t\subseteq [0,\tau_{s})\\ A_{S}\exp[i\omega_{S}t+\theta_{2}]{\vec{e}}_{S}, & \;\;\;\;t\subseteq [\tau_{s},2\tau_{s})\\ \vdots & \\ A_{S}\exp[i\omega_{S}t+\theta_{n}]{\vec{e}}_{S}, & \;\;\;\;t\subseteq [(n-1)\tau_{s},T_{P}) \end{array}\right.$$

where $A_{S}$ is the electric field strength of the target echo light, $\omega_{S}$ is the optical frequency of the target echo, and ${\vec{e}}_{S}$ is the unit vector of the polarization direction of the target echo. $\theta_{i}$ is the phase noise introduced by the change in the target surface topography undulation. For general targets, the change in the target surface topography undulation is much larger than the laser wavelength and $\theta_{i}$ can be considered to be uniformly distributed within [−π,π), and $\langle \theta_{i},\theta_{i+1}\rangle =0$.

Similarly, the light field of the LO light $E_{Lo}(t)$ can be expressed as:

(2) $$E_{Lo}(t)=A_{Lo}\exp[i\omega_{Lo}t]{\vec{e}}_{Lo}$$

where $A_{Lo}$ is the electric field strength of the LO light, $\omega_{Lo}$ is the optical frequency of LO light, and ${\vec{e}}_{Lo}$ is the unit vector of the polarization direction of the LO light.

The polarization direction of the target echo field is matched to the polarization direction of the LO light by a polarizer. In the detection system shown in Figure 1a, the photon flow density $\lambda^{C(D)}(t)$ incident on SPAD(C) and SPAD(D) after the interference between the target echo and the LO light is:

(3) $$\left\{\begin{array}{c} \lambda^{C}(t)=\left[{\left|A_{Lo}\right|}^{2}+{\left|A_{S}\right|}^{2}+2\left|A_{S}A_{Lo}\right|\cos\left(\Delta \omega t+\theta_{M}\right)\right]/2h\nu \\ \lambda^{D}(t)=\left[{\left|A_{Lo}\right|}^{2}+{\left|A_{S}\right|}^{2}+2\left|A_{S}A_{Lo}\right|\cos\left(\Delta \omega t+\pi +\theta_{M}\right)\right]/2h\nu \; \end{array}\right.,t\subseteq [(M-1)\tau_{s},M\tau_{s})$$

where hν is the single photon energy and $\theta_{M}$ is the relative phase between the LO light and the target echo within $\left[(M-1)\tau_{s},M\tau_{s}\right)$.

According to Equation (3), the probability $P^{C}(\Delta t_{i}^{C})$ that the SPAD(C) generates a photon counting plus (PCP) at the moment $T_{M+m}+\Delta t_{i}^{C}\subseteq [(M-1)\tau_{s},M\tau_{s})$ is:

(4) $$\begin{array}{cc} P^{C}(\Delta t_{i}^{C}) & =\eta \lambda^{C}(T_{M+m}+\Delta t_{i}^{C})\times \exp\left(-\int_{0}^{\Delta t_{i}^{C}}\eta \lambda^{C}(T_{M+m}+\xi )d\xi \right)\\ & \approx \frac{\eta }{2h\nu }\left\{{\left|A_{Lo}\right|}^{2}+{\left|A_{S}\right|}^{2}+2\left|A_{S}A_{Lo}\right|\cos\left[\Delta \omega (T_{M+m}+\Delta t_{i}^{C})+\theta_{M}\right]\right\}\\ & \times \exp\left(-\frac{\eta }{2h\nu }\left[{\left|A_{Lo}\right|}^{2}+{\left|A_{S}\right|}^{2}\right]\Delta t_{i}^{C}\right), \end{array}$$

where η is the quantum efficiency of SPAD(C) and $T_{M+m}$ is the enable signal for SPAD(C); the SPAD starts to respond to the photons after $T_{M+m}$. $\Delta t_{i}^{C}\subseteq [0,t_{d})$ is the delay time relative to the $T_{M+m}$ of the generated counting pulse, and $t_{d}$ is the dead time of the detector.

Similarly, the probability $P^{D}(\Delta t_{j}^{D})$ that the SPAD(D) generates a PCP at the moment $T_{M}+\Delta t_{j}^{D}\subseteq [(M-1)\tau_{s},M\tau_{s})$ is:

(5) $$\begin{array}{cc} P^{D}(\Delta t_{j}^{D}) & =\eta \lambda^{D}(T_{M}+\Delta t_{j}^{D})\times \exp\left(-\int_{0}^{\Delta t_{j}^{D}}\eta \lambda^{D}(T_{M}+\xi )d\xi \right)\\ & \approx \frac{\eta }{2h\nu }\left\{{\left|A_{Lo}\right|}^{2}+{\left|A_{S}\right|}^{2}+2\left|A_{S}A_{Lo}\right|\cos\left[\Delta \omega (T_{M}+\Delta t_{j}^{D})+\pi +\theta_{M}\right]\right\}\\ & \times \exp\left(-\frac{\eta }{2h\nu }\left[{\left|A_{Lo}\right|}^{2}+{\left|A_{S}\right|}^{2}\right]\Delta t_{j}^{D}\right), \end{array}$$

Introducing $\Delta t=T_{M+m}+\Delta t_{i}^{C}-(T_{M}+\Delta t_{j}^{D})$, by analyzing Equations (4) and (5), it can be seen that the probability distribution $P_{S}(\Delta t)$ of the time interval between two neighboring PCPs generated by the SPAD(C) and SPAC(D) is:

(6) $$\begin{array}{cc} P_{S}(\Delta t) & =\int_{-\pi }^{\pi }P^{D}(\Delta t_{j}^{D})P^{C}(\Delta t_{i}^{C})\times \exp\left(-\frac{\eta }{2h\nu }\left[{\left|A_{Lo}\right|}^{2}+{\left|A_{S}\right|}^{2}\right](mt_{d}-\Delta t_{j}^{D})\right)d\theta_{M}\\ & \approx \beta \left\{{\left[{\left|A_{Lo}\right|}^{2}+{\left|A_{S}\right|}^{2}\right]}^{2}-2{\left|A_{Lo}A_{S}\right|}^{2}\cos(\Delta \omega \Delta t)\right\}\exp\left[-\frac{\eta }{2h\nu }({\left|A_{Lo}\right|}^{2}+{\left|A_{S}\right|}^{2})\Delta t\right], \end{array}$$

where $\beta =\frac{\eta }{2({\left|A_{Lo}\right|}^{2}+{\left|A_{S}\right|}^{2})h\nu }$ is the normalization factor of the probability.

While for the case of $T_{M}+\Delta t_{j}^{D}\subseteq [(M-1)\tau_{s},M\tau_{s})$ and $T_{M+m}+\Delta t_{i}^{C}⊄[(M-1)\tau_{s},M\tau_{s})$ the probability distribution $P_{N}(\Delta t)$ of the time interval is:

(7) $$\begin{array}{cc} P_{N}(\Delta t) & =\int_{-\pi }^{\pi }P^{D}(\Delta t_{j}^{D})P^{C}(\Delta t_{i}^{C})\times \exp\left(-\frac{\eta }{2h\nu }\left[{\left|A_{Lo}\right|}^{2}+{\left|A_{S}\right|}^{2}\right](mt_{d}-\Delta t_{j}^{D})\right)d\theta_{M}\\ & \approx \beta {\left[{\left|A_{Lo}\right|}^{2}+{\left|A_{S}\right|}^{2}\right]}^{2}\exp\left[-\frac{\eta }{2h\nu }({\left|A_{Lo}\right|}^{2}+{\left|A_{S}\right|}^{2})\Delta t\right], \end{array}$$

Ultimately for the whole process of detection, the total number of time intervals $N_{total}$ detected within $T_{P}$ can be approximated as:

(8) $$\begin{array}{cc} N_{total} & =\left\{\frac{\tau_{s}}{t_{d}}\left[1-\exp(-\frac{\eta }{2h\nu }({\left|A_{Lo}\right|}^{2}+{\left|A_{S}\right|}^{2})t_{d})\right]-1\right\}\frac{T_{P}}{2\tau_{s}}+\left(\frac{T_{P}}{2\tau_{s}}-1\right)\\ & \approx \left\{\frac{\tau_{s}}{t_{d}}\left[1-\exp(-\frac{\eta }{2h\nu }({\left|A_{Lo}\right|}^{2}+{\left|A_{S}\right|}^{2})t_{d})\right]-1\right\}\frac{T_{P}}{2\tau_{s}}, \end{array}$$

where the first term on the right side of the equation is the number of time intervals that follow the $P_{S}(\Delta t)$, and the second term follows the $P_{N}(\Delta t)$. It takes the condition of approximate equivalence as: $\eta \frac{{\left|A_{Lo}\right|}^{2}+{\left|A_{S}\right|}^{2}}{h\nu }\tau_{s}\geq \frac{T_{P}}{\tau_{s}}$.

By analyzing Equations (6) and (8), it can be seen that for the photon time–domain differential detection method, the time interval data are not affected by the fast-varying phase noise $\theta_{m}$. However, for the conventional method of detecting the photocurrent for LHD $\int_{0}^{T_{P}}\left[\lambda^{C}(t)-\lambda^{D}(t)\right]\exp(i\omega_{C}t)dt=\int_{-\pi }^{\pi }\left[\lambda^{C}(t)-\lambda^{D}(t)\right]d\theta_{M}\to 0$, the heterodyne photo-currents cancel each other.

The analysis results show that phase noise causes the photocurrents detected by the conventional LHD method to be discontinuous in the time domain. The photocurrents in different time zones are affected by phase noise with different phases, and ultimately, the photocurrents cannot be effectively accumulated and cancel each other during the whole detection time. It can be seen that there are two main sources of phase noise, firstly, the phase noise generated by atmospheric turbulence and target surface modulation during laser transmission, and secondly, the phase noise generated by the laser itself.

For the phase noise introduced in the laser transmission process, the current research focuses on interfering with LO light by expanding the beam of the target echo using multiple detectors to simultaneously detect photocurrents at each position after beam expansio and. phase noise suppression by post-processing algorithms to compensate for the phase of the heterodyne photocurrent generated by each detector. This scheme is more for the suppression of phase noise caused by the spatial phase of the target echo not coinciding with the LO light [12,18,19,24,25,26,27,28,29]. However, the suppression of phase noise that changes rapidly over time due to rapid changes in the target surface is not satisfactory. The second study is on the suppression of phase noise in the laser itself [30,31,32,33,34], which is also unsatisfactory for photon-level weak target echoes and the lack of prior information on target surface changes.

This is because the effect of phase noise on the detected photocurrent data in the conventional LHD method manifests itself as a multiplicative noise. In the absence of a priori knowledge about phase noise, the filtering effect of multiplicative noise is not satisfactory, whereas in our method, the effect of phase noise on the detected time interval data behaves as additive noise, which can be effectively filtered out in the frequency domain.

According to Equations (7) and (8), the statistical distribution D[{Δt}] of the detected time intervals {Δt} is:

(9) $$D[\{\Delta t\}]=P_{S}(\Delta t)\times \frac{N_{total}}{\tau_{S}}DT+n(\Delta t),$$

where DT is the time jitter of the detector, $\tau_{S}=\frac{2h\nu }{\eta ({\left|A_{Lo}\right|}^{2}+{\left|A_{S}\right|}^{2})}$ characterizes the effective probe length of the time interval data, and n(Δt) is the statistical noise with a mean of 0 and variance $\frac{N_{total}}{\tau_{S}}DT$.

According to Equations (6) and (9), the heterodyne frequency Δω can be obtained by applying the Fourier transform to the detected time interval data {Δt}:

(10) $$\Delta \omega =\underset{\omega_{C}}{\arg\max}\left\{{\left|\frac{1}{N_{total}}\sum_{i=1}^{N_{total}}\exp(i\omega_{C}\Delta t_{i})\right|}^{2}\right\}$$

When $\eta \frac{{\left|A_{Lo}\right|}^{2}+{\left|A_{S}\right|}^{2}}{2h\nu }\tau_{s}\geq \frac{T_{P}}{\tau_{s}}$, ${\left|A_{Lo}\right|}^{2}=2{\left|A_{S}\right|}^{2}$,

(11) $$\begin{array}{cc} SNR & =\eta \frac{{\left|A_{S}\right|}^{2}}{h\nu }T_{P}\frac{{\left|A_{S}\right|}^{2}{\left|A_{Lo}\right|}^{2}}{{\left({\left|A_{S}\right|}^{2}+{\left|A_{Lo}\right|}^{2}\right)}^{2}}\frac{{\left|A_{Lo}\right|}^{2}}{{\left|A_{S}\right|}^{2}+{\left|A_{Lo}\right|}^{2}}\\ & =\frac{4}{27}\eta \frac{{\left|A_{S}\right|}^{2}}{h\nu }T_{P} \end{array}$$

where $\eta \frac{{\left|A_{S}\right|}^{2}}{h\nu }T_{P}$ is the maximum SNR of the conventional heterodyne detection method without the random phase noise of the target echo, while the SNR of the conventional heterodyne detection method is $\eta \frac{{\left|A_{S}\right|}^{2}}{h\nu }\tau_{s}$.

The principle of velocity measurement by heterodyne detection is to obtain the heterodyne spectrum, so the error in the velocity measurement can be characterized by the heterodyne spectrum error std(Δω):

(12) $$std(\Delta \omega )\propto \frac{1}{\sqrt{SNR}}\times \frac{1}{2\tau_{S}}$$

By analyzing Equations (6) and (11), the time interval statistic is a relative quantity, which effectively avoids the effect of random phase noise in LHD. It can be seen that the limitation of the target echo random phase noise in conventional LHD is broken due to the unique photon time–domain differential detection and time-interval data solving method. Our proposed method improves the SNR of LHD under the influence of random phase noise and reduces the velocity measurement error.

## 4. Experiments on Signal-to-Noise Ratio and Velocimetry Error in Detecting the Surface Topographic Undulation of Moving Targets

**Figure 2:**
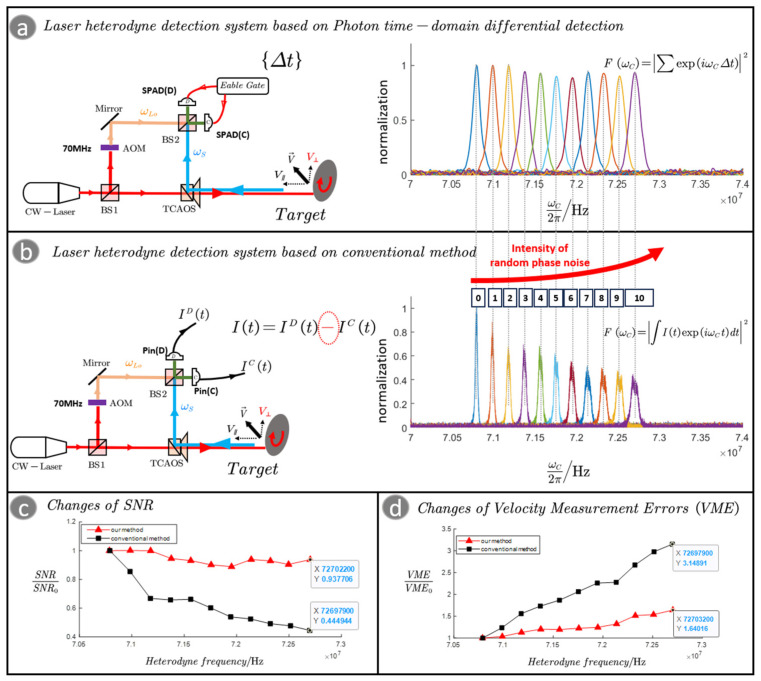
(**a**) The laser heterodyne detection system based on the photon time–domain differential detection. (**b**) The laser heterodyne detection system based on the conventional method by detecting the photocurrent. (**c**) The variation in SNR with heterodyne frequency shift for the conventional method and our method. (**d**) The variation in velocity measurement error with heterodyne frequency shift.

We designed an experimental system to quantitatively analyze and compare the photon time–domain differential detection method with the conventional method. The experimental system is shown in Figure 2a,b; in the experiment, we use a high-speed rotating turntable as the target. The turntable is placed at an angle (45°), and the turntable can be decomposed into a radial velocity and a transverse velocity with respect to the detection beam. As the rotational speed of the turntable increases, the increase in transverse velocity can increase the random phase noise caused by the surface topography undulations, and the increase in radial velocity can increase the Doppler frequency. In this way, we can visually compare the detection results at different phase noise intensities in the same spectrum in the experimental results. In the experimental results of Figure 2a,b, the horizontal coordinate is the frequency in Hz and the vertical coordinate is the normalized spectrum. In the experiment, 200-grit sandpaper is pasted on the surface of the turntable to simulate the surface topography undulation. In the experiment, the rotational speed of the turntable is controlled to control the intensity of random phase noise. The data acquisition time is 0.5 s. The polarization of the laser is controlled to ensure that the target echo is matched with the polarization of the LO light.

For the detection system based on the photon time–domain differential detection shown in Figure 2a, the photon counting rates of the target echo in the experiment are 54 Kcps@SPAD(D) and 44 Kcps@SPAD(C). And the counting rate of the LO light is 80 Kcps@SPAD(D) and 66 Kcps@SPAD(C). We detected the time interval data {Δt} like in Figure 1b and gated the heterodyne spectrum by processing {Δt}. Figure 2b is the LHD system based on the conventional method of detecting the photocurrent for LHD; the power of the LO light is 13 µW. PIN Photodiodes (Pin) is a detector that can linearly respond to light intensity. $I^{C}(t)$ is the photon current generated by Pin(C) and $I^{D}(t)$ is the photon current generated by Pin(D). Finally, the heterodyne spectrum is obtained by processing I(t). Figure 2c shows the variation in SNR with heterodyne frequency shift for the conventional system and our system. Figure 2d shows the variation in velocity measurement error with heterodyne frequency shift.

By analyzing the experimental results shown in Figure 2a,b, we can see that as the rotational speed of the turntable increases, there is an increase in the random phase noise and an increase in the Doppler frequency. In Figure 2c, with the increase in random phase noise, the SNR of the conventional method by detecting the photocurrent gradually decreases to the original value of 0.4, while the SNR of our method decreases to the original value of 0.9, meaning it is almost unaffected. As shown in Figure 2d, the velocity measurement error increases for both methods. However, due to the significant degradation of the SNR, the velocity measurement error of the conventional method increases almost twice as fast as that of our method.

**Figure 3:**
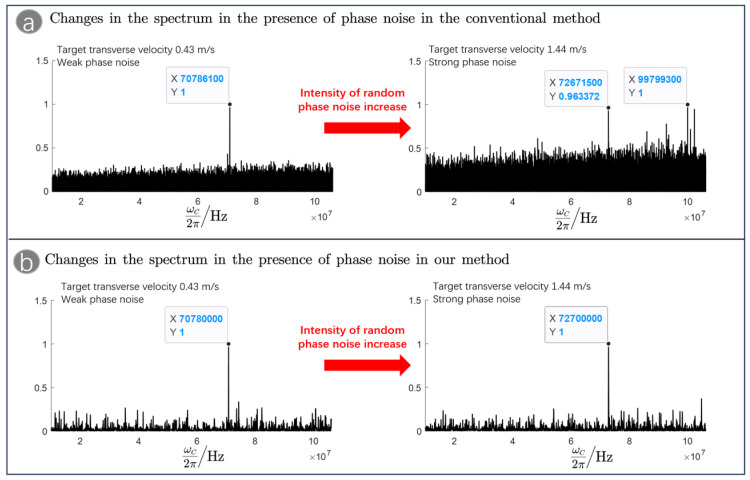
(**a**) Changes in the spectrum of the conventional method in the presence of phase noise conditions and (**b**) changes in the spectrum of our method in the presence of phase noise conditions.

As shown in Figure 3a,b, we compared the spectrograms detected by the conventional method and our method in the presence of phase noise. By analyzing Figure 3a, it can be seen that for the conventional method, the increase in phase noise leads to a significant increase in the noise in the spectrum, and it is not even possible to obtain an accurate heterodyne frequency based on the maximum value of the spectrum, which seriously affects the accurate detection of the target velocity. By analyzing Figure 3b, it can be seen that in our method, the increase in phase noise hardly affects the detected spectrum. This is because our method completes the heterodyne detection by detecting the time interval data, and the time interval data are a relative quantity, which can effectively avoid the accumulation of phase noise in the detection data, thus suppressing the phase noise and achieving the purpose of improving the SNR.

**Figure 4:**
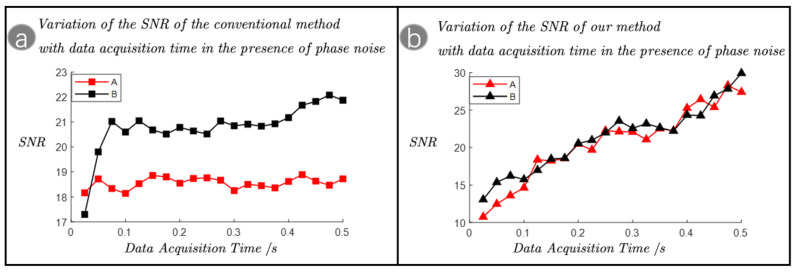
(**a**) Variation of the signal-to-noise ratio of the conventional method with the data acquisition time in the presence of phase noise and (**b**) variation of the signal-to-noise ratio of our method with the data acquisition time in the presence of phase noise. Group A corresponds to a target transverse velocity of 1.44 m/s, which corresponds to the strong phase noise. Group B corresponds to a target transverse velocity of 1.13 m/s, which corresponds to the weak phase noise. The target surface is pasted with 200-grit sandpaper to simulate the rapidly changing phase noise generated by the target surface undulation.

For the detection of weak target echoes at the photon-level, a long data acquisition time is usually required to collect enough echo photons to achieve a sufficient SNR to ensure the accuracy of detection. For this reason, we compared the SNR to the data acquisition time for the conventional method and our method in the presence of phase noise, as shown in Figure 4a,b. In the experiment, group A corresponds to a target transverse velocity of 1.44 m/s, which corresponds to the strong phase noise. Group B corresponds to a target transverse velocity of 1.13 m/s, which corresponds to the weak phase noise. The target surface was pasted with 200-grit sandpaper to simulate the rapidly changing phase noise generated by the target surface undulation.

By analyzing Figure 4a, it can be seen that for the conventional method, when the phase noise is weak corresponding to group B, the SNR firstly increases with the increase in the data acquisition time, but after the SNR is increased to a certain extent, the SNR stays unchanged, and it is difficult to improve the SNR by increasing the data acquisition time. When the phase noise is strong corresponding to group A, the SNR hardly transforms with the data acquisition time. Comparison of the SNR of groups A and B shows that the maximum SNR decreases with increasing phase noise. This is because the phase noise will lead to the discontinuity of the photocurrent detected by the conventional method. And the phase of the photocurrent in different time zones is randomly affected by the phase noise, which means that increasing the data acquisition time does not result in the photocurrent being accumulated efficiently, and most of the photocurrents cancel each other out; therefore, increasing the time for data acquisition has limited impact on the improvement of SNR. As shown in Figure 4b, our method accomplishes heterodyne detection by detecting time intervals, which has the advantage that phase noise can be effectively filtered out by the relative quantity of time intervals, thus effectively avoiding the accumulation of phase noise in the detected data. Even in the presence of phase noise, the SNR still increases linearly with the extension of the data acquisition time. This is especially important for photon-level weak echo detection, which requires a long data acquisition time to obtain a sufficient SNR.

In conclusion, the method proposed in this paper accomplishes heterodyne detection by probing the time interval of photon counting pulses, and its detection SNR and detection error are almost independent of the phase noise of the echo. This means that our method can use poorly coherent lasers for heterodyne detection. Our method can be upgraded on the existing lidar system based on the photon time-of-flight detection of target distance to detect the intensity of the echo and the optical frequency of the echo to realize the direct measurement of the target distance and the target velocity and to enhance the detection dimension of the target echo [6,35,36].

## 5. Conclusions

In this paper, a novel laser heterodyne detection model based on photon time–domain differential detection for time interval data is proposed. The complete theory of obtaining the heterodyne spectrum based on time interval data is established. The time interval statistic is a relative quantity, which can effectively avoid the effect of random phase noise in laser heterodyne detection. Our method can improve the signal-to-noise ratio and reduce the velocity measurement error of laser heterodyne detection in atmospheric turbulence and moving target surface topography irregularities, which is important in LIDAR application scenarios such as synthetic aperture radar and advanced remote sensing. Meanwhile, for photon-level weak echoes that require a longer data acquisition time to be detected, our method can improve the detection signal-to-noise ratio well compared with the conventional heterodyne detection method that detects the photocurrent mode.

Experiments have shown that when the target surface is rough and has transverse motion relative to the laser, fast-changing phase noise is generated in the target echo. Currently, the suppression of this phase noise introduced by the laser during transmission is mainly for the slowly changing phase noise generated by the spatial phase of the target echo that does not coincide with the local oscillator light. The effect is not ideal for suppressing the phase noise that changes rapidly over time due to the rapid change in the target surface. In addition, for this fast-changing phase noise, conventional heterodyne detection methods for detecting and processing photocurrent data cannot effectively improve the signal-to-noise ratio by increasing the data acquisition time, especially for weak target echoes at the photon level. However, our method can linearly improve the signal-to-noise ratio by increasing the data acquisition time, which is a significant advantage for weak echoes at the photon level that require a longer data acquisition time to be detected.

## Data Availability

Data are available upon request due to privacy restrictions.
